# TGF-β1 induces human alveolar epithelial to mesenchymal cell transition (EMT)

**DOI:** 10.1186/1465-9921-6-56

**Published:** 2005-06-09

**Authors:** Hidenori Kasai, Jeremy T Allen, Roger M Mason, Takashi Kamimura, Zhi Zhang

**Affiliations:** 1 Teijin Biomedical Laboratory, Medical Research Council Technology, 1–3 Burtonhole Lane, London, NW7 1AD, UK; 2 Biosciences Research Institute, University of Salford, Greater Manchester M5 4WT, UK; 3 Renal Medicine Section, Faculty of Medicine, Imperial College London, Hammersmith Hospital, London, W12 0NN, UK; 4 Institute for Bio-Medical Research, Teijin Pharma Ltd, Tokyo, 191-8512, Japan

## Abstract

**Background:**

Fibroblastic foci are characteristic features in lung parenchyma of patients with idiopathic pulmonary fibrosis (IPF). They comprise aggregates of mesenchymal cells which underlie sites of unresolved epithelial injury and are associated with progression of fibrosis. However, the cellular origins of these mesenchymal phenotypes remain unclear. We examined whether the potent fibrogenic cytokine TGF-β1 could induce epithelial mesenchymal transition (EMT) in the human alveolar epithelial cell line, A549, and investigated the signaling pathway of TGF-β1-mediated EMT.

**Methods:**

A549 cells were examined for evidence of EMT after treatment with TGF-β1. EMT was assessed by: morphology under phase-contrast microscopy; Western analysis of cell lysates for expression of mesenchymal phenotypic markers including fibronectin EDA (Fn-EDA), and expression of epithelial phenotypic markers including E-cadherin (E-cad). Markers of fibrogenesis, including collagens and connective tissue growth factor (CTGF) were also evaluated by measuring mRNA level using RT-PCR, and protein by immunofluorescence or Western blotting. Signaling pathways for EMT were characterized by Western analysis of cell lysates using monoclonal antibodies to detect phosphorylated Erk1/2 and Smad2 after TGF-β1 treatment in the presence or absence of MEK inhibitors. The role of Smad2 in TGF-β1-mediated EMT was investigated using siRNA.

**Results:**

The data showed that TGF-β1, but not TNF-α or IL-1β, induced A549 cells with an alveolar epithelial type II cell phenotype to undergo EMT in a time-and concentration-dependent manner. The process of EMT was accompanied by morphological alteration and expression of the fibroblast phenotypic markers Fn-EDA and vimentin, concomitant with a downregulation of the epithelial phenotype marker E-cad. Furthermore, cells that had undergone EMT showed enhanced expression of markers of fibrogenesis including collagens type I and III and CTGF. MMP-2 expression was also evidenced. TGF-β1-induced EMT occurred through phosphorylation of Smad2 and was inhibited by Smad2 gene silencing; MEK inhibitors failed to attenuate either EMT-associated Smad2 phosphorylation or the observed phenotypic changes.

**Conclusion:**

Our study shows that TGF-β1 induces A549 alveolar epithelial cells to undergo EMT via Smad2 activation. Our data support the concept of EMT in lung epithelial cells, and suggest the need for further studies to investigate the phenomenon.

## Background

Idiopathic pulmonary fibrosis (IPF), the most common pulmonary fibrotic disorder, is a progressive and lethal disease of unknown etiology whose pathogenesis uniquely features the presence of fibroblastic foci in the parenchyma of the lungs [1]. These are comprised of aggregates of mesenchymal cells including fibroblasts and cells which exhibit phenotypic features of myofibroblasts, α-smooth muscle actin (αSMA) expression, increased mitogenic capacity, and enhanced extracellular matrix (ECM) production. The number of fibroblastic foci correlates with worsening lung function, progression of IPF and a poor prognosis [2]. According to the recent epithelial/fibroblastic model of IPF pathogenesis it is considered that fibroblastic foci underlie areas of unresolved epithelial injury and are sites where activated fibroblasts and myofibroblasts migrate, proliferate and synthesize ECM proteins [3]. However, the cellular origins of the mesenchymal phenotypes in fibroblast foci remain unclear.

It is now well recognized from many studies that a number of key growth factors are responsible for driving the process of fibrogenesis [4]. For example, transforming growth factor-beta1 (TGF-β1), interleukin-1 beta (IL-1β), and tumor necrosis factor-alpha (TNF-α) are able to induce the characteristic motility, proliferation and ECM synthesis observed in mesenchymal cells with a myofibroblast-like phenotype from fibroblastic foci. In general though, it is levels of TGF-β1 that best correlate with the extent of fibrosis and myofibroblast-like cell induction [5] and TGF-β1 continues to be regarded as the most important of the growth factors involved in pulmonary fibrogenesis [6]. For example, the biologically active form of TGF-β1 was aberrantly expressed in the epithelial cells lining honeycomb cysts within the lung of patients with IPF [7,8]. An increased level of TGF-β1 was found in BAL fluid derived from patients suffering from IPF [8]. Furthermore, overexpression of TGF-β1 in lung tissue induced prolonged pulmonary fibrosis in an animal model [9].

Recent evidence from studies of other fibrotic disorders, including renal [10,11] and liver fibrosis [12], supports a view that TGF-β1 may play a novel role in pulmonary fibrogenesis by promoting alveolar epithelial cell transition to form mesenchymal cells with a myofibroblast-like phenotype [10-14]. This process, termed epithelial-mesenchymal transition (EMT), occurs widely under both physiologic and pathologic conditions, for example during normal wound healing [13] and renal fibrosis [10,11]. Very recently it was reported that TGF-β1 induced type II alveolar epithelial cells isolated from rat lung to undergo EMT [15]. Epithelial cells are polarised, and display cytokeratin filaments and membrane-associated junctions. During EMT membrane-associated adherens junctions and desmosomes are dissociated, whilst at the same time or shortly after, cytoskeletal rearrangement takes place and mRNA for intermediate filament proteins is increased, facilitating the cell adopting a mesenchymal phenotype [14]. E-cadherin (E-cad) is an epithelial cell transmembrane protein whose extracellular domain interacts with that of an E-cad molecule expressed by an adjacent cell. It has a critical role in establishing firm adhesion, maintaining cell polarity and epithelial tightness [16]. The cadherin complex suppresses the dissociation of epithelial cells, and thus, the crucial step of EMT is the downregulation of E-cad [14].

To begin to understand the role of EMT in the development of fibroblastic foci in IPF, we have examined whether TGF-β1 can induce EMT in a human lung epithelial cell line (A549). A549 cells retain important characteristics of alveolar type II epithelial cells and have been employed in numerous studies as a valuable tool for studying promoter activity [17], apoptosis [18], and alveolar epithelial cell DNA damage [19]. In this study we demonstrate that TGF-β1 induces EMT in human type II alveolar epithelial cells through the activation of Smad2, and that this transition is accompanied by functional changes that are relevant to the progression of lung fibrosis.

## Methods

### Materials

Recombinant human TGF-β1, human IL-1β; and TNF-α were purchased from R&D systems (Minneapolis, MN). A mouse monoclonal antibody against human E-cad was from BD Transduction Laboratory (Oxford, UK). Mouse monoclonal anti-human fibronectin EDA^+ ^splice form (Fn-EDA) and anti-human cytokeratin-19 were purchased from Abcam Ltd (Cambridge, UK). Goat anti-actin (C-11), Smad2 (S-20) and connective tissue growth factor (CTGF) polyclonal antibodies were supplied by Santa Cruz Biotechnology (Santa Cruz, CA). Rabbit anti-Erk1/2 and rabbit anti-phosphorylated Erk1/2 polyclonal antibodies were purchased from New England Biolabs (Hertfordshire, UK). Both biotinylated and non-biotinylated goat anti-human collagen type I, type III, and type IV polyclonal antibodies, and their standard proteins were supplied by Chemicon International (Temecula, CA). Rabbit anti-mouse immunoglobulin-HRP, rabbit anti-goat immunoglobulin-HRP and goat anti-rabbit immunoglobulin HRP were from DAKO Ltd (Cambridgeshire, UK). Rabbit anti-goat IgG-fluorescein (FITC) was purchased from Vector Labs (Perterborough, UK). Alkaline phosphatase-conjugated anti-Extravidin was supplied by Sigma (Dorset, Poole). PD-098059 and U-0126 were supplied by Merck Bioscience Ltd (Nottingham, UK). SMARTpool SMAD2 and non-specific control pool were purchased from Dharmacon, Inc. (Chicago, IL).

### Cell culture

Human type II alveolar epithelial cells (A549) were supplied from ATCC/LGC Promochem (Middlesex, UK). Cells were maintained in low glucose-DMEM containing 10% FBS, 2 mM L-glutamine, 100 U/ml penicillin and 100 ug/ml streptomycin at 37°C in a humidified 5% CO_2 _atmosphere. Confluent cultures of cells were maintained in serum-free DMEM containing 0.1% BSA for 24 h prior to stimulation with cytokines. The cells were incubated with several concentrations of the cytokines for the periods indicated. In experiments using inhibitors of MAPK/Erk kinase (MEK), the cells were preincubated for 1 h with PD-098059 or U-0126 (up to 10 μM) before treatment with exogenous TGF-β1. In small interfering RNA (siRNA)-dependent gene silencing experiments, after transfection the cells were stimulated with 5 ng/ml of TGF-β1 in serum free 0.1% BSA/DMEM for 48 h. The cells were then harvested and lysed. In experiments testing collagen expression, the cells were incubated in the presence of 5 ug/ml of L-ascorbic acid. For immunocytochemical staining, the cells were maintained throughout the experiment in culture medium without serum deprivation.

### SDS-PAGE and Western blot

The cells were scraped and lysed in M-PER (Pierce, Cheshire, UK) containing a protease inhibitor cocktail (Roche Diagnostic, East Sussex, UK). Cell suspensions were cleared by centrifugation at 13,000 g for 15 min at 4°C. Total protein concentration was measured using the BCA protein assay kit (Pierce) with bovine serum albumin as the standard protein. Equal amounts of protein were loaded for each lane of 10% SDS PAGE gels, followed by electrophoresis, and protein transfers to Hybond ECL membranes (Amersham, Buckinghamshire, UK), as described previously [14]. After the transfer, membranes were blocked with 5% skimmed milk and then probed with primary antibodies for 1 h at room temperature. After washing, the membranes were probed with appropriate peroxidase-conjugated secondary antibodies. After further extensive washing, the immunoblots were visualized by ECL (Amersham) and the band densities for each phenotype marker were quantified using QuantityOne Software (Bio-Rad) after scanning with a GS-710 Calibrated Imaging Densitometer (Bio-Rad). Results were expressed as a ratio of band density to total actin. For Western blotting of phosphorylated proteins, the cells were scraped and lyzed in RIPA buffer containing a protease inhibitor cocktail (Roche), plus 1 mM sodium-orthovanadate and 1 mM NaF. Western blot was performed according to manufacturer's instructions for each antibody. Changes in levels of phosphorylated proteins were assessed with reference to the respective non-phosphorylated proteins.

### Reverse Transcription PCR (RT-PCR)

Total RNA was isolated using Trizol (Invitrogen, Carlsbad, CA). A one-step RT-PCR was performed using the SuperScript One-Step RT-PCR kit (Invitrogen). The target transcript was reverse transcribed at 50°C for 30 min. The PCR products for type III collagen and GAPDH were amplified using 35 cycles (initial denaturation at 94°C/2 min followed by PCR amplification, 94°C/15 s, 60°C/30 s, and 68°C/1 min). For type I collagen, the PCR product was amplified using 35 cycles (initial denaturation at 94°C/2 min followed by PCR amplification, 94°C/30 s, 58°C/30 s, and 68°C/1 min). PCR products were visualized on a GelDoc 1000 system (Bio-Rad) and semi-quantified using a scanning densitometer. The following primers were used for amplification of target transcripts; human collagen type I forward; 5'-ACGTCCTGGTGAAGTTGGTC-3', human collagen type I reverse; 5'-ACCAGGGAAGCCTCTCTCTC-3', human collagen type III forward; 5'-AGCCTCATTAGTCCTGATGGTTCTCG-3', human collagen type III reverse; 5'-CTTCTCAGCACTAGAATCTGTCCACC-3', human GAPDH forward; 5'-GGGCTGCTTTTAACTCTGGT-3', human GAPDH reverse; 5'-TGGCAGGTTTTTCTAGACGG-3'.

### RNA Interference and Transfection

Dharmacon's SMARTpool SMAD2 siRNA reagent includes a pool of 4 SMARTselection-designed synthetic Smad2 siRNA duplexes, together with a non-specific control pool of siRNA as a negative control. Transfection of these pooled 21-nucleotide siRNA duplexes was carried out using Lipofectamine 2000 (Invitrogen), following the manufacturer's instructions. Cells were seeded at 5 × 10^4 ^cells/well of 24 wells plate and then incubated in normal medium without antibiotics for overnight to reach 80–90% confluence. siRNA-transfection reagent complexes were prepared as described by the manufacturer. Briefly 1 μl/well Lipofectamine 2000 was mixed with either 50 pmol/well Smad2 or negative control siRNA. FITC conjugated dsRNA (Block-iT, Invitrogen) was used as positive control. The cells were then transfected with siRNA/Lipofectamine complexes in Opti-MEM (Invitrogen) and incubated for 24 h at 37°C in a CO_2 _incubator. Following incubation, transfection efficiency was evaluated under fluorescence microscopy.

### Immunocytochemistry

Cells were plated into a Lab-TeK Chamber Slide at a density of 2 × 10^4 ^cells/well and grown until approximately 80% confluent, after which they were treated with TGF-β1 at 5 ng/ml for 72 h, as described above. The cells were washed, fixed in ice-cold methanol for 10 min, and then soaked in PBS containing 2% TritonX-100 for 5 min to increase their permeability to antibodies. These cells were exposed to antibodies (1:20) for 1 h. After washing, bound primary antibodies were detected using appropriate FITC-conjugated secondary antibodies (1:50). Images were collected using an Eclipse TE2000-S microscope system (Nikon UK Ltd, Surrey) and Image-Pro Plus (Media Cybernetics UK, Berkshire).

### Gelatin zymography for matrix metalloproteinases (MMPs) expression

Conditioned media were concentrated approximately 10-fold using ultra-centrifugation. After measuring protein concentration, equal amounts of samples were mixed with an equal volume of 2 × non-reducing SDS PAGE sample buffer. The samples were applied to a 10% (w/v) polyacrylamide gel impregnated with 2 mg/ml gelatin (Sigma). After electrophoresis, SDS was removed from the gel by washing 3 times for 10 min in 2.5% Triton X-100 solution. Then the gels were incubated overnight with gentle shaking at 37°C in buffer (50 mM Tris-HCl (pH7.6), 10 mM CaCl2, 50 mM NaCl, 0.05% Brij35), after which, the gel was stained with 0.25% Coomassie blue R250 in 40% methanol and 10% acetic acid for 2 h at room temperature, and subsequently destained with a 40% methanol-10% acetic acid solution until the bands became clear.

### ELISA

Culture media were analyzed for collagens type I, type III and type IV using sandwich ELISA [14]. Each purified human collagen was used as a standard. Culture media were incubated in ELISA plates in which wells had been coated with non-biotinylated primary antibodies. Following addition of biotinylated antibodies, the plates were washed and reacted with alkaline phosphatase-conjugated anti-Extravidin. P-nitrophenyl phosphate substrate tablets were used to detect alkaline phosphatase activity and the product was measured at 405 nm using a micro-plate reader (Bio-Rad).

### Statistical analysis

Data are presented as the mean ± SD of at least three independent experiments. For statistical analysis, an unpaired t-test was used for pair-wise comparisons and ANOVA with Dunnett's post test for other data. Statistical analysis was performed using commercial statistical software Prism Version 4.0 (GraphPad, Software Inc., San Diego, CA). P values less than 0.05 were considered as statistically significant.

## Results

### TGF-β1 induces alveolar epithelial cells to undergo EMT

**Figure 1:**
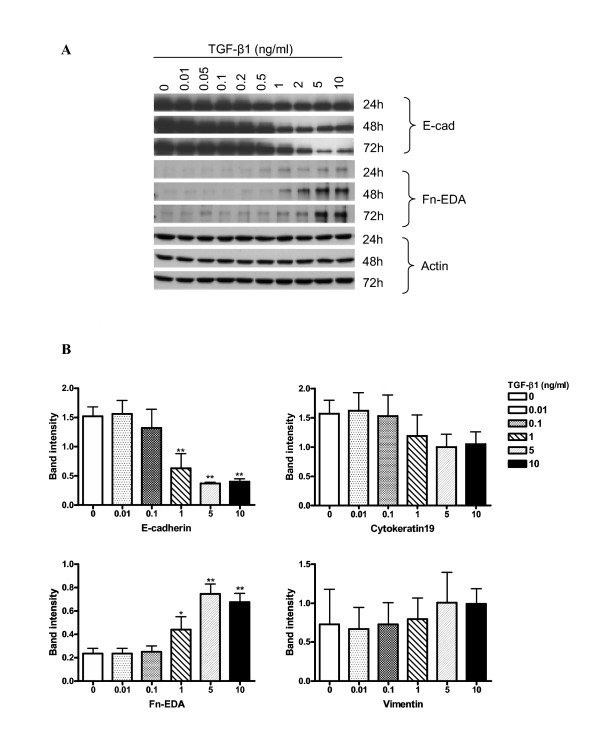
**Expression changes of EMT-related markers in A549 cells. **(A) A549 cells were incubated with up to 10 ng/ml of TGF-β1 in the absence of serum for up to 72 h. Expression of the epithelial marker E-cadherin is down-regulated by TGF-β1 stimulation in a concentration-and time-dependent manner. Expression of Fn-EDA, which is a mesenchymal marker, is up-regulated by TGF-β1 in parallel with the down regulation in the epithelial marker. The same amounts of total protein are loaded in each lane. (B) Densitometric analysis of band intensities for each EMT related marker was performed at 48 h. Each bar represents mean ± SD of three independent experiments. * P < 0.05 and ** P < 0.01.

**Figure 2:**
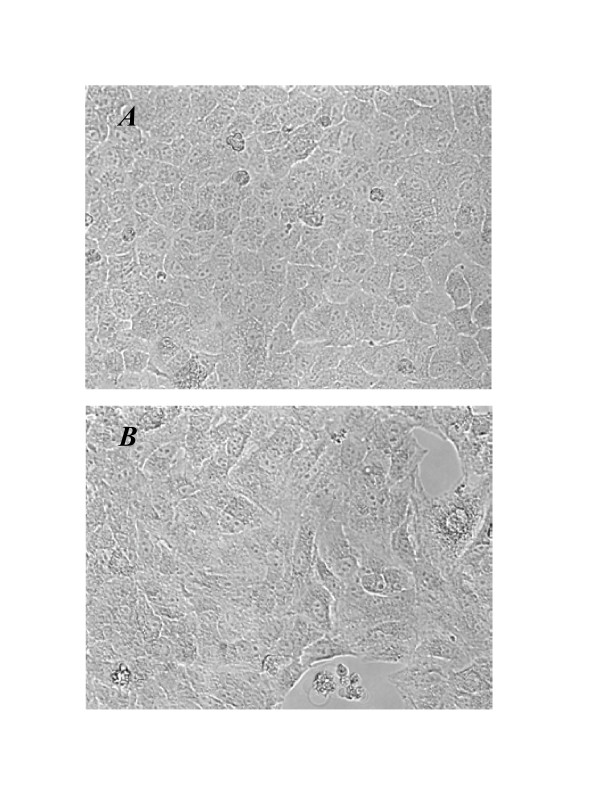
**Morphological changes induced by TGF-β1. **A549 cells were incubated with 5 ng/ml of TGF-β1 for 48 h. (A) Untreated A549 cells show a pebble-like shape and cell-cell adhesion is clearly observed. (B) TGF-β1-treated cells show a decrease in cell-cell contacts and adopt a more elongated morphological shape (magnification of 200×).

We first determined the optimum concentrations and time required for TGF-β1 to initiate EMT in cultures of the alveolar type II epithelial cell line, A549. The expression of the epithelial phenotype markers, E-cad and cytokeratin 19, and of the mesenchymal phenotype markers, Fn-EDA and vimentin, were determined following treatment of A549 cells with various concentrations (0.01–10 ng/ml) of TGF-β1 for 24, 48 and 72 h (Figure 1). Changes in cell morphology were also assessed under phase contrast light microscopy (Figure 2).

TGF-β1 significantly (P < 0.01) decreased E-cad expression in a concentration-and time-dependent manner (Figure 1A). Concentrations as low as 1 ng/ml of TGF-β1 induced up to 70% loss of E-cad expression within 48 h (Figure 1B). However, the extent of cytokeration 19 suppression was not as profound as for E-cad. Only high concentrations of TGF-β1 resulted in a measurable decrease (Figure 1B). In parallel with the marked decrease in the E-cad epithelial marker, TGF-β1 significantly (P < 0.01) induced expression of the mesenchymal marker Fn-EDA, in a concentration-and time-dependent manner (Figure 1A). But the level of vimentin expression was not as profound as for Fn-EDA. Our data also suggested that the *de novo *expression of Fn-EDA might occur earlier than E-cad suppression (Figure 1A).

In addition to the changes in the phenotypic markers expressed in A549 cells after TGF-β1 stimulation, the cells also underwent morphological changes on exposure to the growth factor (Figure 2). A549 cells cultured in the absence of TGF-β1 maintained a classic cobblestone epithelial morphology and growth pattern (Figure 2A), but after stimulation with 5 ng/ml of TGF-β1 for 48 h, the cells adopted a more fibroblast-like morphology and reduced their cell-cell contact (Figure 2B).

**Figure 3:**
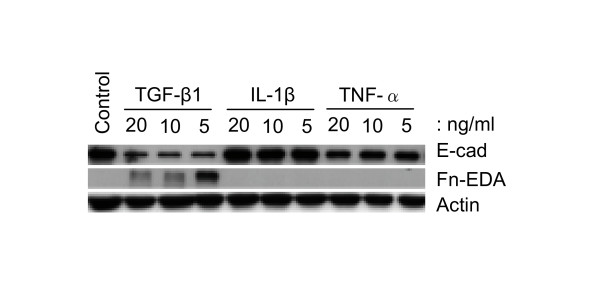
**Comparison of EMT-related marker expression in response to TGF-β1, IL-1β and TNF-α treatments. **A549 cells were incubated with TGF-β1, IL-1β and TNF-α at the indicated concentrations for 48 h. Only TGF-β1 decreases E-cadherin expression concomitant with increasing Fn-EDA expression. In contrast, TNF-α only slightly decreases E-cadherin expression and IL-1β has no influence on EMT-related marker expression. Equal amounts of total protein are loaded in each lane.

Both IL-1β and TNF-α have been suggested to play a role in fibroblast/myofibroblast motility, proliferation and ECM synthesis [4], and IL-1β induces kidney epithelial cells to undergo EMT [20,21]. We therefore tested whether these cytokines had similar properties to TGF-β1 in inducing lung alveolar epithelial cells to form mesenchymal-like cells. Up to 20 ng/ml of IL-1β had no effect on the expression of any of the molecular markers examined, whilst TNF-α, at 20 ng/ml concentration, resulted in a ~20% fall in the expression of E-cad (Figure 3). Neither TNF-α nor IL-1β induced the expression of Fn-EDA. These results suggested that alveolar EMT might be induced by aberrant expression and activation of TGF-β1 rather than by IL-1β or TNF-α.

### TGF-β1-induced EMT is accompanied by other changes relevant to fibrogenesis

**Figure 4:**
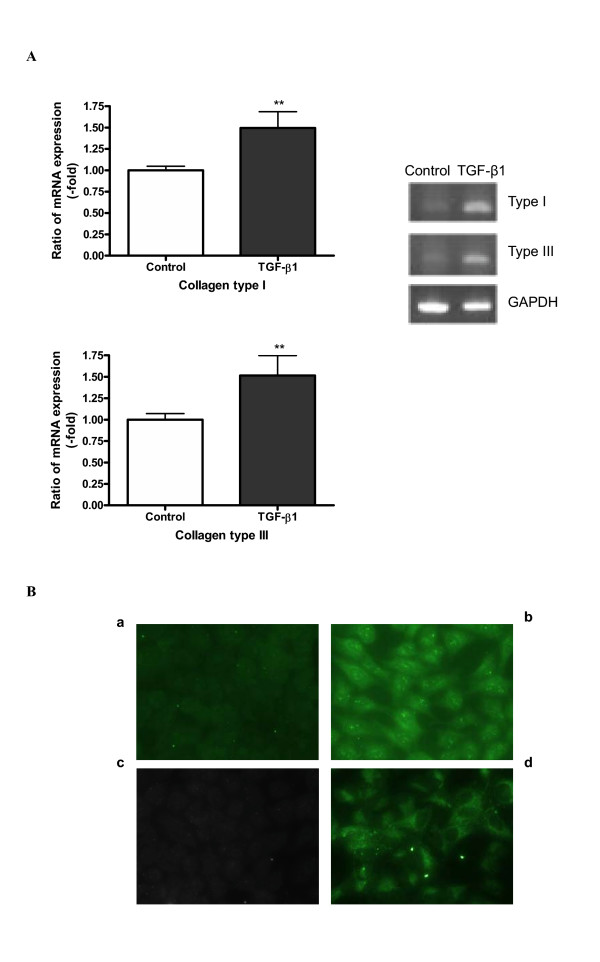
**TGF-β1 induces the expression of collagens type I and type III. **A549 cells were incubated with 5 ng/ml of TGF-β1 in the presence of 5 μg/ml of L-ascorbic acid for 72 h. (A) mRNA expression of collagens type I and type III was detected using RT-PCR. Densitometric analysis was performed. The changes of expression level are expressed as fold increase compared to the control. Each bar represents the mean ± SD of three independent experiments. ** P < 0.01. (B) Protein expression of collagens type I and type III was detected by immunocytochemical staining. Panels (a) and (b) represent collagen type I and panels (c) and (d) represent collagen type III expression, respectively. TGF-β1 induces collagen type I and type III expression in A549 as shown in panel (b) and (d).

**Figure 5:**
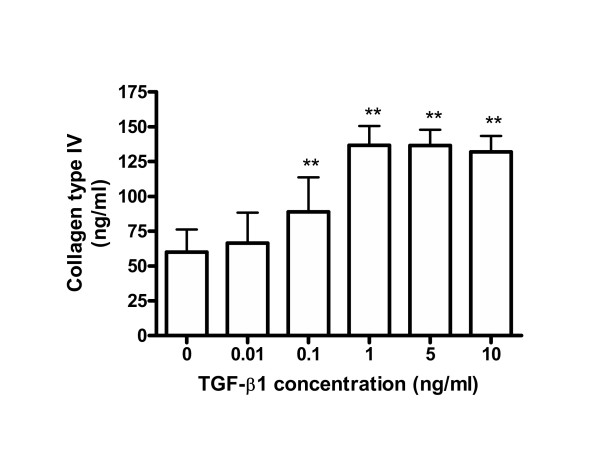
**TGF-β1 increases collagen type IV secretion in A549. **A549 cells were incubated with several concentrations of TGF-β1 in the presence of 5 μg/ml of L-ascorbic acid for 72 h. Concentrations of collagen type IV in conditioned media were determined using ELISA. TGF-β1 increases collagen type IV in a concentration-dependent manner. Each bar was expressed as the mean ± SD of four independent experiments. ** P < 0.01.

The results presented above suggest that lung epithelial cells take on a mesenchymal-like phenotype in response to TGF-β1. To confirm this, we examined the expression of collagen type I and type III by A549 cells in the presence and absence of TGF-β1, as these fibrillar collagens are characteristically synthesized by fibroblastic-type cells. We compared the production of these fibrillar collagens with that of type IV collagen which is characteristically synthesized by epithelial cells making basement membrane. Cells were treated with several concentrations of TGF-β1, and the effects on collagen expression assessed by RT-PCR; protein synthesis of collagens was also examined by ELISA and immunocytochemical staining. Figure 4 shows that TGF-β1 significantly (P < 0.01) stimulated the expression of collagens type I and type III, as detected by both RT-PCR (Figure 4A) and immuno-staining of the cell layer (Figure 4B). Protein levels of collagens type I and type III secreted into the culture medium were too low to be measured quantitatively by ELISA. Interestingly, the secretion of collagen type IV into the medium was increased in the presence of TGF-β1 (Figure 5). Concentrations of TGF-β1 as low as 0.1 ng/ml significantly induced secretion of collagen type IV as compared with control cells, the increased secretion level reaching a plateau at 1 ng/ml of TGF-β1 (Figure 5).

**Figure 6:**
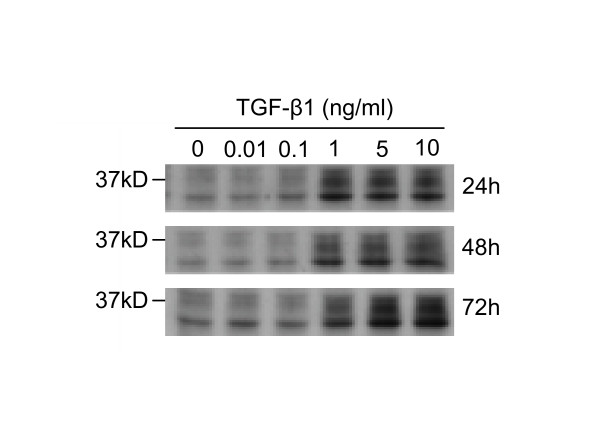
**TGF-β1 induces CTGF expression in A549. **A549 cells were incubated for the times indicated with the concentrations of TGF-β1 shown. Cell lysates were used as a source for Western blot analysis of CTGF expression. Up-regulation of CTGF expression is observed with 1 ng/ml and higher concentrations of TGF-β1. This phenomenon parallels the altered expression of EMT related markers. Representative blots are shown from three independent experiments.

CTGF acts in concert with TGF-β1 and is thought to have a significant role in promoting and maintaining fibrogenesis [22]. Thus, we investigated whether the expression of CTGF in A549 was affected by TGF-β1 treatment. Western analysis of both A549 cell lysates (Figure 6) and culture medium (data not shown) indicated that treatment with TGF-β1 at concentrations >1 ng/ml upregulated the expression of native CTGF (36–38 kDa) in a time-dependent manner. An additional smaller immunoreactive CTGF species was also detected and may correspond to a CTGF breakdown product which is frequently found in cell cultures [23].

**Figure 7:**
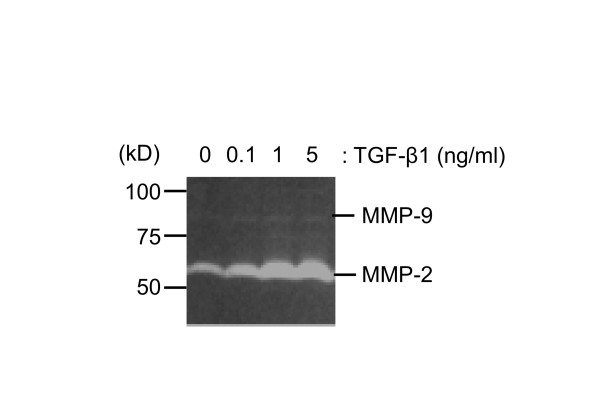
**Effect of TGF-β1 on MMPs expression in A549. **Gelatin zymography was performed using the conditioned media that were harvested after 48 h TGF-β1 treatment (0.1 to 5 ng/ml). The samples were applied without reduction to a 10% polyacrylamide gel containing gelatin, and proteolytic activity was demonstrated by digestion of the gelatin and clearing of the gel.

MMPs expression was examined using gelatin zymography to check whether A549 cells adopt characteristics necessary for cell migration in response to TGF-β1 treatment. As shown in Figure 7, A549 cells express gelatinases with molecular weights consistent with an identity of MMP-2 and MMP-9. MMP-2 was the main gelatinase expressed and TGF-β1 treatment up-regulated MMP-2 expression in a concentration-dependent manner. Basal MMP-9 expression was low and TGF-β1 had almost no effect on it.

### EMT is associated with TGF-β1 signalling through the Smad pathway rather than via MAP Kinases

**Figure 8:**
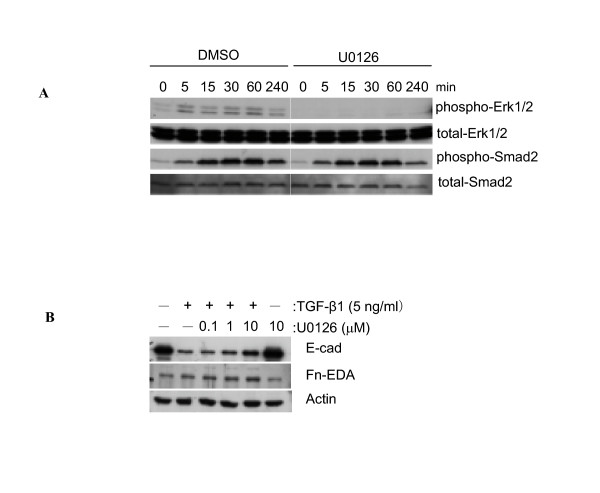
**Activation of Smad and ERK1/2 pathways by TGF-β1. **A549 cells were pre-incubated in the presence or the absence of 10 μM of U0126, a potent MEK inhibitor, for 1 h prior to TGF-β1 stimulation. 5 ng/ml of TGF-β1 was used as a stimulus. TGF-β1 activates Erk1/2 and Smad2 pathways within 5 min after stimulation. The MEK inhibitor blocks Erk1/2 phosphorylation, but does not influence Smad2 phosphorylation. Equal amounts of total protein are loaded in each lane.

TGF-β1 signaling involves both the Smads and MAP kinases pathways [24]. The phosphorylation of Erk1/2 and Smad2 was examined at various time points after adding TGF-β1 (5 ng/ml) to A549 cells. The phosphorylation of Erk1/2 was increased slightly at 5 min after stimulation, and this effect lasted for at least 4 h without alteration of total Erk1/2 protein (Figure 8A). The TGF-β1-induced phosphorylation of Erk1/2 was completely suppressed in the presence of the MEK inhibitors, PD98059 (data not shown) or U0126 (Figure 8A). However MEK inhibitors had little or no effect on TGFβ1-induced changes in the expression of EMT markers over 48 h (Figure 8B).

Interestingly, 5 ng/ml of TGF-β1 induced phosphorylation of Smad2 within 5 min of stimulation, and the level of Smad2 phosphorylation reached a maximum between 30–60 min after treatment and remained elevated for the duration of the experiment without affecting total Smad2 expression (Figure 8A). Co-incubation with either of the MEK inhibitors, PD98059 (data not shown) or U0126, had no effect on the TGF-β1 mediated Smad2 phosphorylation (Figure 8A). Taken together, these data indicate that rapid and sustained phosphorylation of Smad2 is associated with TGF-β1-induced EMT events and that TGF-β1-induced Erk1/2 signalling pathways are less likely to be involved in the EMT of A549 cells.

### siRNA-mediated Smad2 gene silencing inhibits TGF-β-mediated EMT

**Figure 9:**
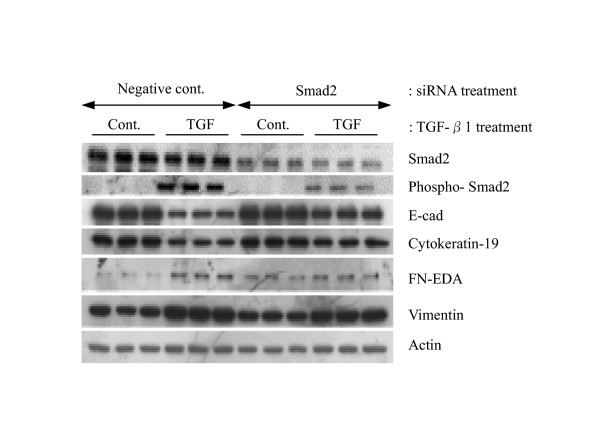
**Effect of Smad2 siRNA on TGF-β1 induced EMT in A549 cells. **Pooled synthetic siRNA duplexes targeting different regions of Smad2 were transfected into A549 cells at 50 pmol per well. 24 h after transfection, cells were stimulated with 5 ng/ml of TGF-β1 in serum free 0.1% BSA/DMEM for a further 48 h prior to harvest. Equal amounts of lysates were resolved by SDS-PAGE and analyzed by Western blotting for expression of proteins.

In order to confirm whether Smad2 is involved in TGF-β1-mediated EMT, siRNAs were used to silence Smad2 gene expression in A549 cells. Transfection efficiency, using FITC-conjugated dsRNA oligomers, was approximately 100 % (data not shown). Western analysis revealed that total Smad2 protein expression was significantly (P < 0.01) depleted by Smad2 siRNA gene silencing, while pooled negative control siRNA had no detectable effect on Smad2 protein expression (Figure 9). Using a phospho-specific antibody against Smad2, we also tested for the effect of Smad2 siRNA on phosphorylated Smad2 levels after TGF-β1 treatment. In the presence of TGF-β1 phosphorylated Smad2 levels were significantly (P < 0.01) diminished by Smad2 siRNA (Figure 9). Most importantly, Smad2 siRNA restored the decreased expression of E-cad and cytokeration 19 induced by TGF-β1 treatment. However, the expression of Fn-EDA and vimentin were only slightly suppressed by Smad2 siRNA (Figure 9). Nevertheless, our data suggest that the activation of Smad2 signaling pathway is involved in TGF-β1-mediated EMT in A549 cells.

## Discussion

Fibroblastic foci in the IPF lung parenchyma are characterized by vigorous replication of mesenchymal cells, including fibroblasts and myofibroblasts, and subsequent abnormal deposition of ECM proteins [25]. However, precise mechanisms responsible for the formation of fibroblastic foci are unknown and their cellular origins are unclear. It is recognized though, that injury to the alveolar epithelium precedes their formation and that epithelial cells may therefore contribute to their formation, perhaps by their responses to, or production of, key fibrogenic mediators including TGF-β1 [3]. Thus, one possible role of TGF-β1 in IPF is to induce or promote epithelial cells in injured alveolar epithelium to undergo EMT [15], as is evident from studies of other fibrotic disorders such as renal fibrosis [10,11,14]. Cells that have undergone EMT would then contribute to the development of fibroblastic foci and to their abnormal ECM production. The results of our present *in vitro *study support the concept underpinning this hypothesis; namely that TGF-β1 can induce alveolar EMT in human lung epithelial cells via the Smad2 pathway.

Epithelial phenotype is determined by the specific range of proteins expressed by the epithelial cell and the nature of its environment [14,26,27]. E-cad is an epithelial cell transmembrane protein with conserved cadherin repeats in the extracellular domain. In the presence of Ca^2+^, the extracellular domain binds to that of E-cad on an adjacent epithelial cell to form tight cell-cell adhesion and to suppress the dissociation of epithelial cells from their location. Certain morphogenic and/or environmental cues, such as local expression of TGF-β1, result in the loss of epithelial cell polarity, adherens junctions, tight junctions, desmosomes, cytokeratin intermediate filaments and, subsequently, rearrangement of F-actin stress fibers and the development of filopodia and lamelopodia [10,11]. Several *in vitro *studies have demonstrated that addition of TGF-β1 to cultured human epithelial cells from organs other than lung induces them to downregulate E-cad expression and to become mesenchymal cells, resembling myofibroblasts, via EMT [27-29].

In the present study, we investigated the potential for alveolar EMT by examining the expression of phenotypic markers in A549 cells. Although submerged monolayer culture of A549 may not completely mimic the pulmonary epithelium, these cells are still widely used for studies on the role of human alveolar type II epithelial cells as they retain features and metabolic properties characteristic of type II cells [17,19,30]. The present study demonstrates that low doses of TGF-β1 induce A549 cells to lose expression of epithelial phenotypic markers, such as E-cad and cytokeratin 19 expression, similar to our previously reported findings in kidney epithelial EMT [14]. Concurrently A549 cells gain a mesenchymal phenotype with *de novo *expression of Fn-EDA and increased expression of vimentin. In addition to these classic mesenchymal markers, these newly formed mesenchymal cells expressed the fibrillar collagens type I and type III which are likely to contribute to excessive accumulation of ECM in fibrotic tissue. Moreover, these cells expressed an elevated level of MMP-2 after EMT which could contribute to breakdown of basement membranes and facilitate migration. Our data is consistent with the findings made in in kidney tubular epithelial cells where MMP-2 and MMP-9 were up-regulated by TGF-β1 in parallel with changes in EMT markers [27,31]. It remains uncertain whether these fibroblast-like cells possess migratory and/or invasive capabilities, but TGF-β1 induced MMP-2 expression in A549 supports this notion. Clearly further investigation of cell migration in TGF-β1-mediated alveolar EMT in lung fibrosis is warranted.

We have yet to confirm our results in primary human type II alveolar epithelial cells because cultures of these cells rapidly adopt fibroblast-like morphology, even in the absence of TGF-β1. Therefore, we cannot rule out at this stage the possibility that the responses of A549 cells to TGF-β1 are unique to this human cell line. However, a recent report indicates that rat primary type II alveolar epithelial cells undergo EMT *in vitro *in response to treatment of TGF-β1 [15], suggesting EMT is likely to be a phenomenon common to all type II alveolar epithelial cells.

In addition to TGF-β1, many other cytokines, including IL-1β and TNF-α, have been suggested to play a role in IPF [4]. We examined whether, like TGF-β1, IL-1β and TNF-α also convert A549 to fibroblast-like cells. It is widely accepted that IL-1β induces EMT of renal epithelial cells through a TGF-β1-dependent mechanism [20,21]. However, our results showed that both TNFα and IL-1β failed to induce alveolar epithelial cells to undergo EMT, possibly due to the differences in the cell types investigated. Similarly A549 cells failed to respond to stimulation with 30% (v/v) activated PBMC-conditioned medium (aPBMC-CM) (H. Kasai, unpublished data), a stimulus which induces renal epithelial cells to undergo EMT-mediated conversion to myofibroblasts [14,32,33].

We observed that A549 EMT was not accompanied by expression of αSMA, even during longer periods (7 days) of exposure to TGF-β1 (data not shown). Although αSMA, together with vimentin and desmin, is often used to classify myofibroblasts [34,35], the expression of these cellular markers is varied and dependent on cell types and culture conditions [36].

TGF-β1 regulates various cell functions, such as cell proliferation, cell differentiation, apoptosis, cell adhesion/motility, ECM production, and its association with pulmonary fibrosis is well known [37]. *In vivo *studies have demonstrated increased TGF-β1 gene expression and protein secretion in the lungs of animals [38] and humans with fibrotic diseases [7,8,39]. Furthermore, transient overexpression of active TGF-β1 in rat lung resulted in severe interstitial and pleural fibrosis characterized by extensive deposition of ECM proteins, and by the emergence of cells with the myofibroblast phenotype [9]. A time-dependent production of endogenous TGF-β1 from rat alveolar epithelial cells after exogenous TGF-β1 treatment was also noted by Yao *et al *[15], suggesting initial EMT induced by TGF-β1 may result in further EMT induced by endogenous TGF-β1 production in an autocrine or paracrine manner. Based on these studies and our data it is tempting to speculate about a role for TGF-β1-mediated EMT in pulmonary fibrogenesis. Indirectly, our data showing TGF-β1-mediated induction of collagen type I and type III expression, which are present in fibrotic lesions *in vivo *[40], and the expression of Fn-EDA and vimentin which represent cellular markers for myofibroblasts, supports such a role for EMT.

TGF-β1 exerts its effects through heteromeric receptor complexes composed of type I and type II serine/threonine receptors. Upon ligand binding, the type II receptor phosphorylates the type I receptor inducing its kinase activity [41]. TGF-β1 activity may be transduced along the Smads pathway immediately downstream of the receptor complex in a variety of cell systems and also via JNK, p38 MAPK and Erk pathways [37,41,42]. Thus, we examined the intracellular signalling pathway involved in TGF-β1-mediated alveolar EMT. Although phosphorylation and activation of p38 MAPK and Erk1/2 were observed in some lung fibroblasts [43], our data suggested that the signalling pathway involved in alveolar EMT was likely to be a Smad2-dependent pathway since the MEK inhibitors, PD98059 and U0126, failed to reverse TGF-β1-induced phenotypic modulation of A549 cells, whereas Smad2 siRNA attenuated the loss of E-cad and cytokeratin 19 induced by TGF-β1. Smad2 phosphorylation has been noted in EMT processes for several cell types including breast and renal epithelial cells [44-46].

Transition from the alveolar epithelial phenotype to the mesenchymal phenotype initiated by TGF-β1 was accompanied by elevated expression of CTGF. CTGF induction is mediated through a TGF-β1 response element in the CTGF promoter and its mediation of at least some of the activities attributed to TGF-β1 is well recognized [4,22], as is its involvement in the maintenance of fibrogenesis. In lung for example, Allen *et al *reported that the mRNA level of CTGF in BALF cells of patients with IPF was significantly higher than that in healthy control subjects [46]. Furthermore, expression of CTGF was found in both interstitial fibroblasts and type II alveolar epithelial cells in patients with IPF [47]. CTGF secreted by alveolar epithelial cells and myofibroblasts responding to TGF-β1 may also act as a paracrine factor for lung fibroblasts and is known to be a critical intermediate for the synthesis of connective tissue proteins stimulated by TGF-β1, but not by other fibrogenic cytokines [48,49]. CTGF may therefore play a role in mediating the expression of collagens by EMT cells. Most recently, investigations with mesangial cells showed that CTGF interacts with the TrkA receptor, triggering events which lead to the induction of a transcriptional repressor, TIEG [50]. It was proposed that since this suppresses negative regulation of the TGF-β-Smad signaling pathway by repressing Smad7 expression, it leads to enhanced TGF-β1 signaling [51]. It is presently unclear whether CTGF plays a similar role in alveolar epithelial responses to TGF-β1, or in EMT cells derived from them. However, in spite of uncertainties surrounding its precise role, CTGF remains implicated in the pathogenesis of many fibrotic disorders [4], and is likely to contribute significantly to fibrogenesis in the lung.

## Conclusion

Our findings show that TGF-β1 induces an EMT-like process in A549 alveolar epithelial cells, most likely by activation of the Smad2 signaling pathway. These data provide evidence to support the concept that human lung epithelial cells can undergo EMT and indicate a need for further studies. In particular, the expression profile associated with alveolar epithelial cells that have undergone EMT indicates a potential role for EMT in pulmonary fibrogenesis.

## List of abbreviations

IPF = Idiopathic pulmonary fibrosis; αSMA = α-smooth muscle actin; ECM = extracellular matrix; TGF-β1 = transforming growth factor-beta1; IL-1β = interleukin-1 beta; TNF-α = tumor necrosis factor-alpha; EMT = epithelial mesenchymal transition; E-cad = E-cadherin; Fn-EDA = fibronectin EDA^+ ^splice form; CTGF = connective tissue growth factor; MEK = MAPK/Erk kinase; RT-PCR = Reverse Transcription PCR; MMP = matrix metalloproteinase; siRNA = small interfering RNA

## Authors' contributions

HK carried out the cellular and biochemical studies and participated in drafting the manuscript. JA, RM and TK participated in the design of the study and drafted the manuscript. ZZ conceived the study, and participated in its design and coordination, and in drafting and finalizing the manuscript. All authors read and approved the final manuscript.
